# The Impact of Malignancy on Assisted Reproductive Outcomes for Cancer Survivors: A Retrospective Case–Control Study

**DOI:** 10.3389/fonc.2022.941797

**Published:** 2022-09-16

**Authors:** Yuehong Li, Xuefen Cai, Binhua Dong, Qi Wang, Xiaohui Yang, Aili Yu, Huijuan Wei, Zhanghong Ke, Pengming Sun, Beihong Zheng, Yan Sun

**Affiliations:** ^1^ College of Clinical Medicine for Obstetrics & Gynecology and Pediatrics, Fujian Medical University, Fuzhou, China; ^2^ Center of Reproductive Medicine, Fujian Maternity and Child Health Hospital, College of Clinical Medicine for Obstetrics & Gynecology and Pediatrics, Fujian Medical University, Fuzhou, China; ^3^ Laboratory of Gynecologic Oncology, Department of Gynecology, Fujian Maternity and Child Health Hospital, College of Clinical Medicine for Obstetrics & Gynecology and Pediatrics, Fujian Medical University, Fuzhou, China; ^4^ Fujian Key Laboratory of Women and Children’s Critical Diseases Research, Fujian Maternity and Child Health Hospital, College of Clinical Medicine for Obstetrics & Gynecology and Pediatrics, Fujian Medical University, Fuzhou, China; ^5^ Fujian Province Key Laboratory of Environment and Health, School of Public Health, Fujian Medical University, Fuzhou, China

**Keywords:** cancer survivors, infertility, oncofertility, long-term reproductive outcomes, assisted reproductive, ovarian stimulation, pregnancy outcome

## Abstract

**Background:**

Related studies have shown that it is safe for cancer patients to undergo assisted reproduction. However, studies on whether a history of cancer affects long-term reproductive outcomes in women who undergo assisted reproductive technology (ART) are scarce. In this study, we evaluated the long-term reproductive outcomes of patients with malignant tumors undergoing ART treatment and explored the impact of malignancy history on ART outcomes.

**Methods:**

This retrospective study analyzed the clinical outcomes of patients with malignant tumors undergoing their first *in vitro* fertilization/intracytoplasmic sperm injection (IVF/ICSI) cycles compared with those of age-matched healthy infertile women at Fujian Maternity and Child Health Hospital between January 2003 and October 2020. We evaluated ovarian stimulation outcome, the pregnancy rate, the live birth rate, the risk of adverse obstetric outcomes and birth outcomes.

**Results:**

This study included 59 patients in the cancer group for data analysis who had a history of malignancy. By matching, a total of 118 healthy infertile women were included in the control group. No statistically significant association was found in terms of age, duration of infertility, BMI, or insemination type between the two groups of patients. Thyroid cancer(45.8%) and gynecologic malignancies (44.07%) were the major cancer types in this study. There were statistically significant differences in the antral follicle count (AFC) (12.00 ± 7.86 vs. 14.90 ± 8.71, P=0.033), length of ovarian stimulation (9.98 ± 2.68 vs. 11.42 ± 2.43, P=0.033) and endometrial thickness on the trigger day (10.16 ± 3.11 vs. 10.84 ± 2.17, P<0.001) between the two groups. The total gonadotropin dose, number of oocytes retrieved, fertilization rate, cleavage rate, high-quality embryo rate, blastocyst rate and first-time embryo-transfer (ET) implantation rate were nonsignificantly lower in the cancer group than in the control group (P>0.05). There were no significant differences in the clinical pregnancy rate per ET cycle (32% vs. 40.39%, P=0.156), live birth rate per ET cycle (27% vs. 35.96%, P=0.119), miscarriage rate per ET cycle (5% vs. 4.43%, P=0.779), or preterm delivery rate per ET cycle (11.11% vs. 17.80%, P=0.547) between the two groups. Additionally, regression analysis showed that a history of malignancy was not a risk factor for reproductive outcomes.

**Conclusions:**

Overall, it is feasible for women with a history of cancer to conceive using ART is feasible and their long-term reproductive outcomes are similar to these of healthy infertile women. A history of cancer does not decrease the number of retrieved oocytes, increase the risk of adverse obstetric outcomes or affect birth outcomes.

## Introduction

Thousands of women of reproductive age are diagnosed with cancer every year (1). However, the overall survival rate of patients with cancer has improved in recent years (2). Given that the trend toward delayed childbearing is rising, many young patients diagnosed with cancer have not started or completed their families (3). The irreversible cytotoxic effect of cancer therapy on fertility is well known. Oncological treatment interventions, including surgery for gynecological malignancies (4), chemotherapeutic agents (5, 6), abdominal or pelvic radiotherapy (6, 7) and hormonotherapy (8), usually cause infertility, premature ovarian failure or early menopause due to massive destruction of the ovarian reserve (5). Although most young female cancer survivors resume menstruation after the completion of chemotherapy and endocrine therapy, ongoing menstrual function is not the same as normal fecundity (9, 10). Among such patients, reproductive potential might be lost, negatively impacting quality of life (QOL) (11). Thus, with increasing numbers of long-term survivors, fertility and pregnancy issues have become important concerns for these young women.

Today, an increasing number of patients are undergoing treatment with assisted reproductive technology (ART). Diverse fertility preservation options, such as embryo cryopreservation, oocyte cryopreservation, immature oocyte cryopreservation, ovarian tissue cryopreservation and transplantation, have been established for cancer survivors. In addition, ovarian protection with gonadotropin-releasing hormone agonists (GnRH-a) to preserve ovarian function during chemotherapy has been investigated in several randomized trials (12, 13). The American Society of Clinical Oncology (14) (ASCO) and the European Society for Medical Oncology (15) (ESMO) recommend that fertility consultation be provided for all patients of reproductive age who are diagnosed with cancer with prompt referral to fertility specialists. Despite international guidelines, the fertility information offered by oncology specialists is quite sparse (16). Because of the scarcity of relevant research in the literature and insufficient information, it is difficult to provide appropriate counseling to for these patients.

Accumulated research has shown that pregnancy after ART in cancer survivors is safe and that the prognosis does not seem to be affected, but to date, few studies have evaluated the long-term reproductive outcomes of ART among cancer survivors (17–19). Recent studies have shown that women with a history of cancer have a greater risk of several adverse pregnancy outcomes (20). In addition, due to the limited number of cancer patients undergoing ART, most reported studies involve small cohorts and are retrospective studies, and although data from a few multicenter studies are available, prospective studies are lacking. Therefore, knowledge of long-term reproductive and survival outcomes after ART among patients with a history of cancer remains limited, and further study is needed.

We conducted this retrospective case–control study to determine the possible impact of a history of malignant disease on pregnancy outcomes among cancer patients who undergo ART by comparing their results to those of infertile women without a prior cancer diagnosis. This cohort contained detailed registry-based information on maternal infertility, ovarian stimulation, obstetric outcomes and birth outcomes.

## Materials and methods

### Patient selection

This retrospective study screened women with malignant tumors (cancer group) who underwent their first IVF/ICSI treatment at Fujian Maternity and Child Health Hospital between January 2003 and October 2020. Infertile women with nonmalignant tumors who first underwent *in vitro* fertilization/intracytoplasmic sperm injection (IVF/ICSI) during the same period were included in the control group and were age-matched at a ratio of 1:2. The age difference between each pair of cases was no more than 1 year, and the time of transvaginal ultrasound-guided oocyte retrieval was restricted to within the same month. There was no difference in the ART procedures between the two groups. All ART procedures in the cancer group were performed after the completion of cancer treatment.

The inclusion criteria of the control group were as follows: Infertile women with nonmalignant tumors who were undergoing IVF/ICSI for the first time. The exclusion criteria were as follows: patients with a history of recurrent spontaneous abortion or chromosomal abnormalities related to pregnancy outcomes requiring preimplantation genetic testing; patients with malformations of the reproductive system; or patients with incomplete data.

All patient information were collected from hospital records or through phone interviews by phone; specifically, details regarding history of maternal infertility, ovarian stimulation, and pregnancy outcomes and complications were obtained. The cancer treatment history of cancer patients was based on their surgical records and discharge results. The last follow-up of the entire cohort was on May 31, 2021.

### ART procedures

All patients was selected an appropriate ovarian stimulation protocol according to ovarian reserve. Ovarian stimulation was performed using the following protocols: follicular-phase GnRH agonist, luteal-phase GnRH agonist, antagonist, mild stimulus, progestin-primed ovarian stimulation (PPOS) and natural cycle.

In the follicular phase GnRH agonist regimen, we administered 3.75 mg of GnRH-a on days 2–5 of menstruation. After 28 days, if the downregulation standard was reached, gonadotropin (Gn) was started to induce ovulation, and the dose of Gn was adjusted according to follicle growth and serum hormone levels. Downregulation standards were classified as follows: (a) a serum luteinizing hormone (LH) level of <5 IU/L, a follicle-stimulating hormone (FSH) level of <5 IU/L, an estradiol (E2) level of <50pg/ml, and a progesterone (P) level of <1 ng/mL; and (b) no functional cyst, a follicle size of 3–5 mm under ultrasound, and induced ovulation. In the luteal phase GnRH-a protocol, patients received oral contraceptives from day 3 of menstruation for 21 days and then then subcutaneously administered 0.1 mg of GnRH-a daily from day 18 of menstruation until the human chorionic gonadotropin (hCG) trigger day. In the GnRH antagonist regimen, ovarian stimulation was initiated from days 2-3 of menstruation with intramuscular injections of follicle stimulating hormone at a dose of 150–300 IU/day until the hCG trigger day. The dose of gonadotropin was adjusted during the stimulation process according to follicular development, which was determined by ultrasound and serum hormone levels, up to a maximum of 300 IU/day. A daily dose of 0.25 mg of a GnRH antagonist was initiated when a dominant follicle reached a mean diameter of 14 mm or when blood LH levels began to show a notable upward trend; the dose was continued until the day of hCG administration. In the PPOS regimen, patients received oral medroxyprogesterone acetate 8 mg/day and human menopausal gonadotropin (HMG) or a urinary FSH intramuscular injection at 150–300 IU/day from day 2–3 of menstruation until the hCG trigger day. In the mild stimulus regimen, patients received oral letrozole 5 mg/day consecutive five days from days 2–5 of menstruation and HMG intramuscular injection at 75–150 IU/day until the hCG trigger day. The dose of Gn was adjusted during the stimulation process according to the follicle growth and serum hormone levels.

Final oocyte maturation was triggered by hCG when the maximum follicle reached 18 mm in diameter, and transvaginal ultrasound-guided oocyte retrieval was performed 36 hours after the hCG injection. The patient chose IVF/ICSI according to her husband’s semen condition. After 3 days of *in vitro* culture, day-3 embryos were transferred or vitrified; the remaining embryos were cultured to the blastocyst stage after informed consent was obtained from the patient. All transferred or frozen embryos were scored as good-quality embryos, and luteal support was given after transplantation. Embryo quality was assessed according to the criteria established by the Istanbul Consensus Workshop on Embryo Assessment (21). The types of embryo transfer included fresh and frozen–thawed embryo transfers.

### Study outcomes

The ovarian stimulation outcomes of women who underwent their first IVF/ICSI cycles were analyzed, and all frozen–thawed cycles resulting from the embryos preserved from the initial stimulation were included in the analysis. Pregnancy outcomes and adverse pregnancy outcomes were analyzed for all clinical pregnancies after embryo transfer cycles. The incidence of pregnancy complications, including premature rupture of fetal membranes (PROM), gestational diabetes mellitus (GDM) and thyroid disorders in pregnancy, was evaluated.

Clinical pregnancy was defined as at least 1 gestational sac in the uterus identified on by ultrasonography 35 days after embryo transfer. Live birth was defined as the delivery of at least one newborn who exhibited any sign of life, irrespective of gestational duration. Preterm delivery was defined as the delivery of a living fetus before 37 weeks of gestation. Low birth weight (LBW) was defined as a birth weight <2.5 kg. Large for gestational age (LGA) was defined as a birth weight > 4.0 kg.

### Statistical analysis

Data were collected and analyzed using SPSS 23.0 software (SPSS Inc., Chicago, Ill., USA). Quantitative data with a normal distribution are described as the mean ± standard deviation, and those with a nonnormal distribution is described as the median (interquartile range). We used the t test or Wilcoxon rank-sum test to evaluate the distribution of data. Qualitative data are described as the frequency and percentage, and the chi-square test was used to compare the composition of qualitative variables without a causal association. Cox proportional hazard regression and logistic regression were conducted to calculate hazard ratios (HRs) or odds ratios (ORs) with 95% confidence intervals (CIs) to adjust for relevant factors. All statistical tests were 2-sided with a significance level of 0.05.

### Ethics approval

The investigation was conducted in accordance with the ethical standards and the Declaration of Helsinki and according to national and international guidelines. As this was a retrospective study, this study was exempted from ethics approval and approved by the Ethics Committee of Fujian Maternity and Child Health Hospital, College of Clinical Medicine for Obstetrics & Gynecology and Pediatrics, Fujian Medical University (2022KYLLR01027).

## Results

### Patient characteristics

**Figure 1:**
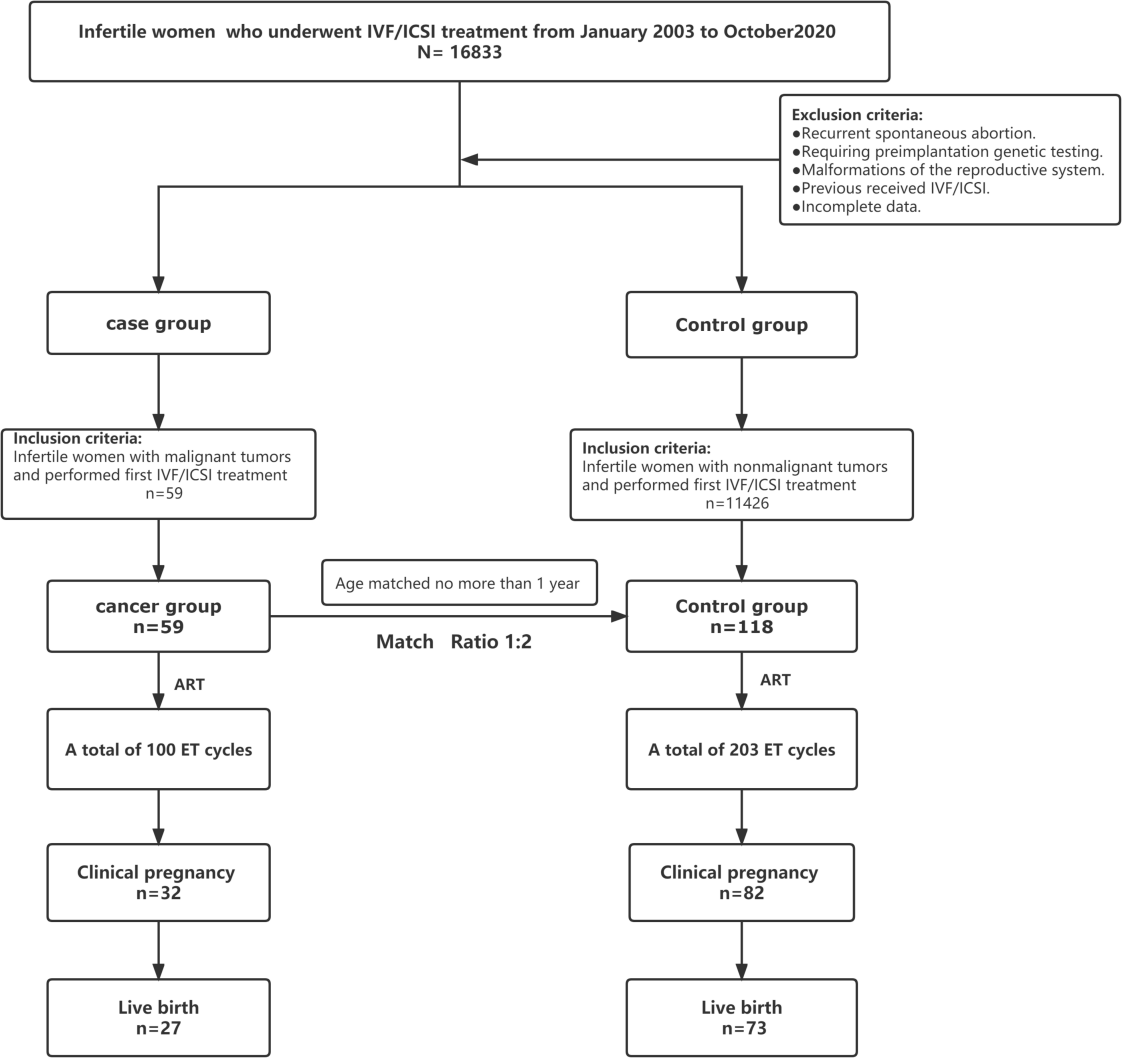
Study diagram.

The recruitment of patients in the trial is reported in **Figure 1**. In total, 16,833 patients with infertility who were referred to the reproductive medicine center of Fujian Maternity and Child Health Hospital for *in vitro* fertilization and embryo transfer (IVF-ET) between January 2003 and October 2020 were screened for eligibility. We included 59 patients who had a history of malignancy in the cancer group for data analysis. Altogether, a total of 118 healthy infertile women identified from patients without a history of tumors were included in the control group. All patients with malignant tumors completed tumor-related treatment, and the median time from the completion of tumor-related treatments to the initiation of assisted reproduction treatment was approximately 36 months (**Table 1**). In the whole cohort, all patients were alive, and no signs of malignant disease occurrence were found in either group at the end of follow-up.

**Table 1:** Patients’ characteristics.

	cancer group(*n*=59)	control group(*n*=118)	*P-value*
Age (year), mean (SD), years	35.75±4.98	35.56±4.76	0.809
Duration of infertility, mean (SD), years	4.29±3.21	3.62±2.67	0.144
Body mass inde (BMI), mean (SD)(kg/m^2^)	21.77±3.22	21.78±2.99	0.982
***Ovarian Reserve***			
Basal FSH, mean (SD), (mIU/ml)	7.30±2.33	6.61±2.13	0.052
Basal LH, mean (SD), (mIU/ml)	4.05±2.69	4.00±2.13	0.886
Basal Estradiol, mean (SD), (pg/ml)	48.98±45.03	41.98±20.48	0.157
Basal Testosterone, mean (SD), (ng/dl)	0.32±0.13	0.33±0.13	0.725
AFC, mean (SD)	12.00±7.86	14.90±8.71	0.033
(n=125) AMH, mean (SD), ng/dl	4.17±3.75	3.64±2.83	0.432
***Tumor type n(%)***			
Thyroid cancer	27 (45.76)	-	-
Female reproductive system tumors*	26(44.07)	-	-
others*	6 (10.17)	-	-
***Treatment manner n(%)***			
surgery	50 (84.75)	-	-
Other treatment manners*	9 (15.25)	-	-
***Type of protocol for controlled-ovarian stimulation***			
Luteal phase GnRH agonist	5 (8.48)	34 (28.81)	<0.001
Follicular phase GnRH agonist	19 (32.20)	51 (43.22)	
Antagonist	13 (22.03)	24 (20.34)	
Mild stimulus	15 (25.42)	4 (3.39)	
Progestin-primed Ovarian Stimulation (PPOS)	4 (6.78)	5 (4.24)	
Natural cycle	3 (5.09)	0 (0.00)	
***Insemination type***			
IVF	46 (77.97)	85 (72.03)	0.396
ICSI	13 (22.03)	33 (27.97)	
Median time M (P_25_, P_75_)*	36.00 (24.00, 84.00)	–	–

FSH, follicle-stimulating hormone; LH, luteinizing hormone; AFC, antral follicle count in both ovaries; AMH, anti-Müllerian hormone; IVF, in vitro fertilization; ICSI, intracytoplasmic sperm injection;

Female reproductive system tumors*18 cases of borderline ovarian tumors, 5 cases of ovarian cancer, 2 cases of endometrial cancer, 1 case of cervical cancer.

others* 2 cases of liver cancer, 1 case of breast cancer, 1 case of nasopharyngeal cancer, 1 case of gastric cancer, 1 case of lymphadenoma.

Other treatment manners*:One patient with nasopharyngeal cancer received surgery and chemoradiotherapy, four patients with ovarian cancer and one patient with borderline ovarian tumors received surgery and chemotherapy, one patient with lymphadenoma received chemotherapy, and two patient with endometrial cancer received dilatation and curettage combined with hormone therapy.

Median time M (P25, P75)*:Median time from completed tumor-related treatments to begin underwent assisted reproduction treatment (month), Interquartile range M (P25,P75).

The patient characteristics of the cohort at baseline are presented in **Table 1**. The main cancer diagnoses in the cancer group were thyroid cancer (45.8%) and female reproductive system malignancies (44.07%), and most patients underwent surgery (84.7%). No statistically significant association was found in terms of the duration of infertility, BMI, basal hormone levels (FSH, LH, estradiol, testosterone) and anti-Müllerian hormone (AMH) levels between the two groups of patients. However, as expected, a lower antral follicle count (AFC) was observed in the cancer group than in the control group (12.00 ± 7.86 vs. 14.90 ± 8.71, P=0.033). Remarkably, the antagonist protocol and mild stimulus protocol were used more frequently in the cancer group than in the control group.

### The first IVF/ICSI cycle outcome

Analysis of the first-cycle ovarian stimulation outcome showed that the dose of gonadotrophins received (2018.20 ± 829.18 vs. 2492.79 ± 918.32, P=0.482) and the length of ovarian stimulation (9.98 ± 2.68 vs. 11.42 ± 2.43, P=0.033) were lower in the cancer group than in the control group (**Table 2**). Additionally, compared to the control group,the cancer group had a significantly lower FSH level (12.52 ± 4.17 vs. 13.35 ± 4.84, P=0.006) and a thinner endometrium (10.16 ± 3.11 vs. 10.84 ± 2.17, P<0.001) on the trigger day (**Table 2**). Nevertheless, there was no statistically significant difference in the estradiol or LH level on the trigger day between the two groups (P>0.05)(**Table 2**). We observed that the number of oocytes retrieved, fertilization rate, cleavage rate, high-quality embryo rate, blastocyst rate, and first-time embryo-transfer implantation rate were lower in the cancer group than in the control group, but the difference was not statistically significant (P>0.05)(**Table 2**). The cancer group had a shorter length of ovarian stimulation than the control group; however, except for the number of first-time embryos transferred, the first IVF/ICSI cycle outcomes were not significantly different between the two groups (1.59 ± 0.50 vs. 1.76 ± 0.43, P=0.031) (**Table 2**).

**Table 2:** The first IVF /ICSI cycle outcomes.

	Cancer group (*n*=59)	Control group (*n*=118)	*P-value*
Total dose of gonadotropins, mean (SD),(IU)	2018.20± 829.18	2492.79 ± 918.32	0.482
Length of Stimulation, mean (SD),(day)	9.98 ± 2.68	11.42 ± 2.43	0.033
***The level of hormone on HCG trigger day***			
FSH, mean (SD),(mIU/ml)	12.52± 4.17	13.35 ± 4.84	0.006
LH ,mean (SD),(mIU/ml)	2.67± 2.53	1.44 ± 1.46	0.407
Estradiol , mean (SD),(pg/ml)	2665.44 ± 2198.25	2980.14 ± 2458.71	0.134
Progesterone, mean (SD),(ng/ml)	0.79 ± 0.48	0.82 ± 0.51	0.001
Endoendometrial thickness on HCG trigger day, mean (SD), (mm)	10.16 ± 3.11	10.84 ± 2.17	<0.001
No. of retrieved oocytes, mean (SD),No.	9 ± 7	10.14 ± 6.67	0.291
Fertilization rate, mean (SD)	0.79 ± 0.27	0.81 ± 0.19	0.665
Cleavage rate, mean (SD)	0.87 ± 0.25	0.92 ± 0.16	0.211
High-quality embryos rate,mean (SD)	0.51 ± 0.32	0.57 ± 0.27	0.187
Blastocyst rate, mean (SD)	0.24± 0.25	0.25± 0.25	0.784
***First-time embryo transfer status***			
(n=165) First-ET number of transferred embryos ,mean (SD)	1.59± 0.50	1.76 ± 0.43	0.031
(n=165) First- ET implantation rate	0.34 ± 0.42	0.36 ± 0.42	0.826
(n=165) First-ET clinical pregnancy rate	0.38 (20/53)	0.49 (55/112)	0.171

GnRH, gonadotrophin-releasing hormone; HCG, human chorionic gonadotropin; FSH follicle-stimulating hormone, LH luteinizing hormone; ET, embryo transfer.

### Assisted reproductive outcomes

The results of the patients with malignant tumors undergoing assisted reproduction were similar to those of the healthy infertile women undergoing ART. A total of 303 transplant cycles were evaluated in this study; the cancer group had a total of 100 embryo transfer (ET)cycles and the control group had a total of 203 ET cycles. As of May 31, 2021, all the tumor patients who were followed up were alive and did not have tumor recurrence. Among the 177 study subjects followed, 114 were clinically pregnant, of which 82 were in the control group and 32 were in the cancer group; 100 babies were successfully delivered, including 73 in the control group and 27 in the case group. There was no significant difference in the number of IVF/ICSI cycles per live birth, the number of embryos transferred per live birth, the number of embryos per live birth or the number of high-quality embryos required per live birth between the two groups (**Table 3**). Moreover, there were no significant differences in the rate of clinical pregnancy per ET cycle (32% vs. 40.39%, P=0.156), live birth per ET cycle(27% vs. 35.96%, P=0.119), miscarriage per ET cycle (5% vs. 4.43%, P=0.779), or preterm birth (11.11% vs. 17.80%, P=0.547) between the two groups (**Table 3**).

**Table 3:** Reproductive outcome.

	Cancer group(*n*=27)	Control group (*n*=73)	*P-value*
No. IVF/ICSI per live birth	1.46± 0.93	1.51 ± 1.25	0.869
No. embryos transferred per live birth	1.46 ± 0.93	1.54 ± 1.16	0.754
No. embryos required per live birth	2.07 ± 1.47	2.66 ± 2.23	0.206
No. high-quality embryos required per live birth	2.04 ± 1.51	2.51± 2.18	0.304
No. clinical pregnancy per ET cycle	32/100	82/203	0.156
No. live birth per ET cycle	27/100	73/203	0.119
No. miscarriage per ET cycle	5/100	9/203	0.779
No. preterm birth per ET cycle	3/27	13/73	0.547

IVF, in vitro fertilization; ICSI, intracytoplasmic sperm injection; ET, embryo transfer.

### Effects of diagnosis of malignancy on pregnancy

#### Influencing factors of pregnancy outcome

**Figure 2:**
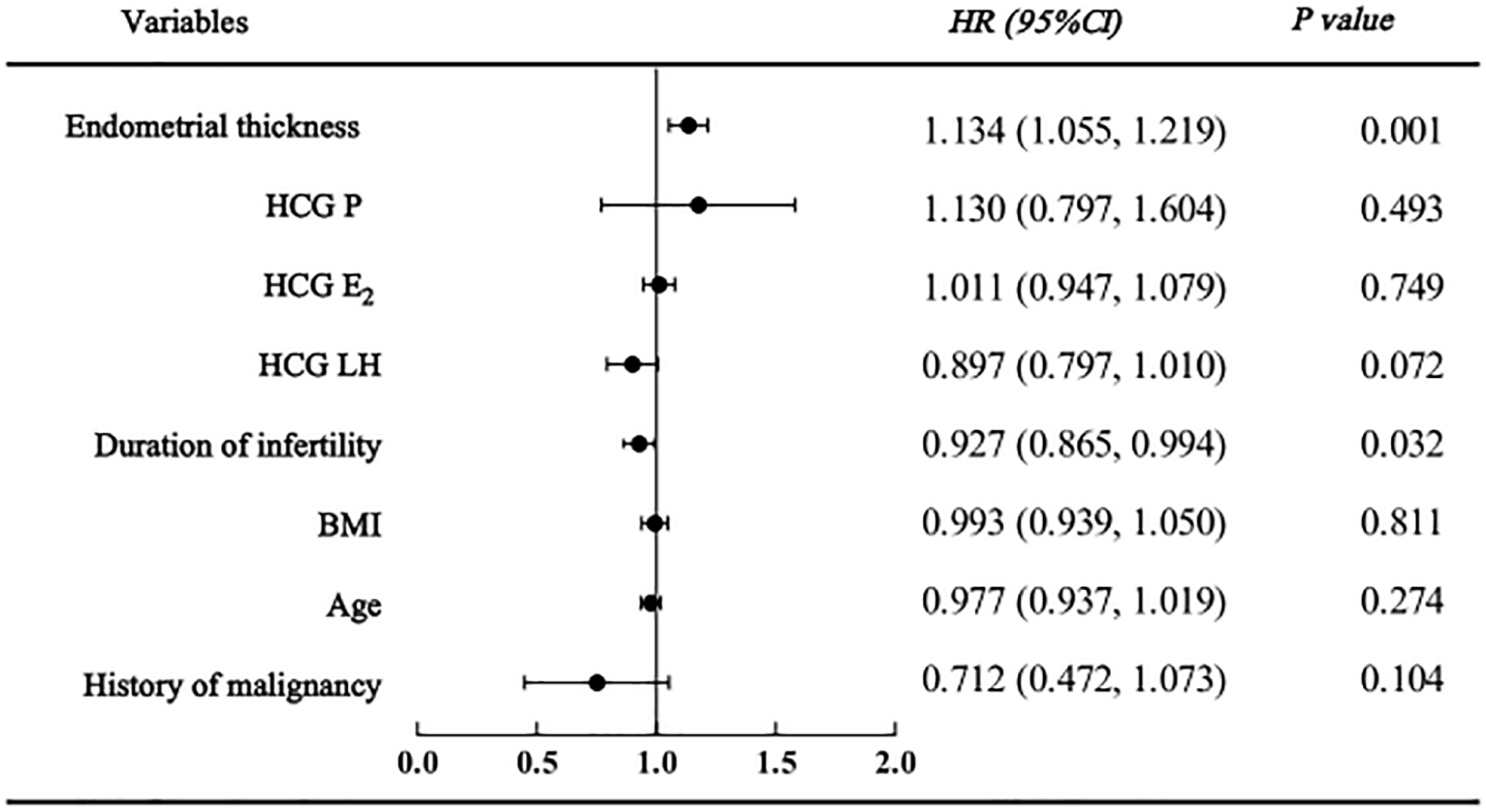
Cox regression analysis forest plot of clinical pregnancy affecting factors.

**Figure 3:**
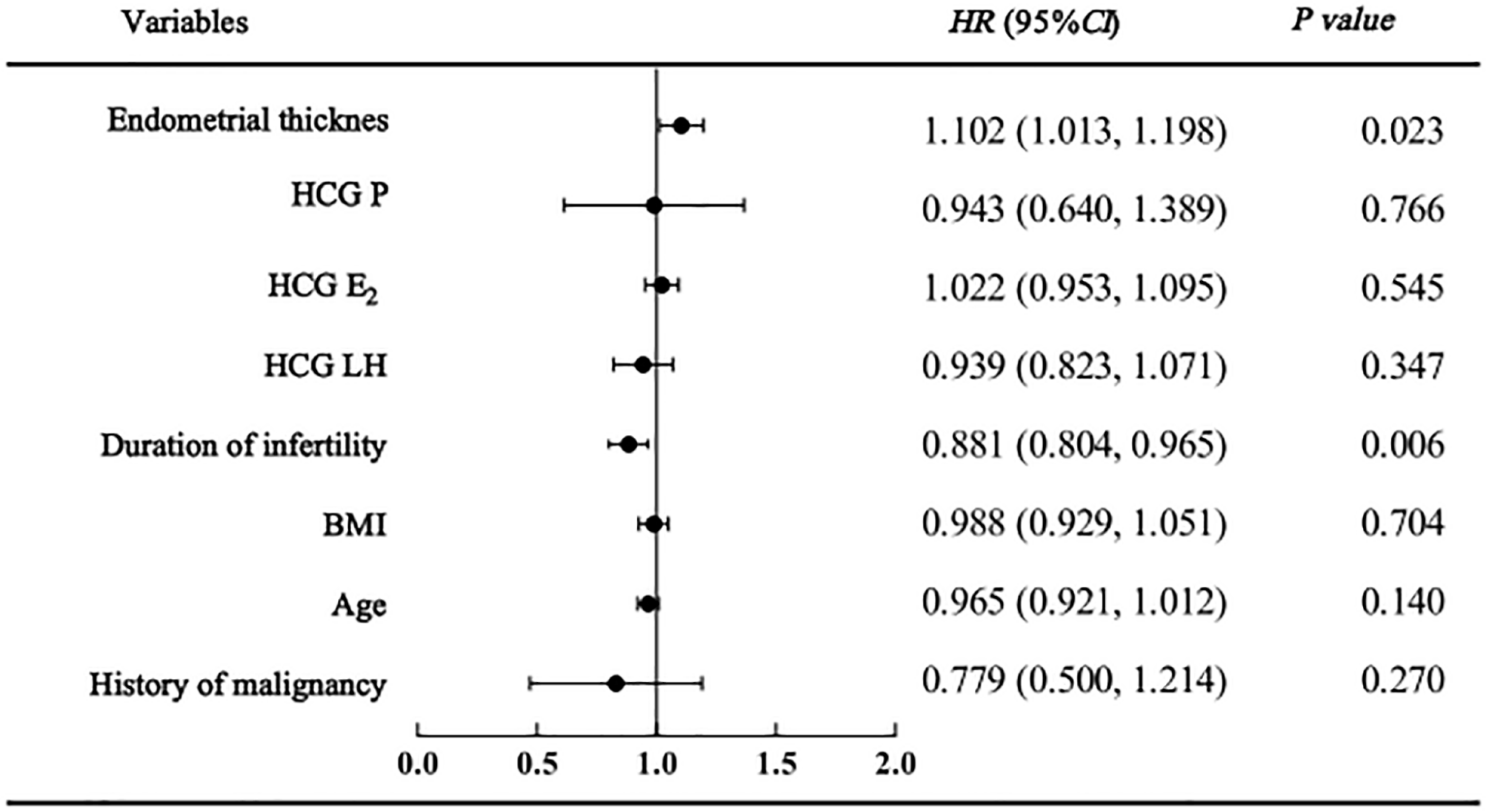
Cox regression analysis forest plot of live birth affecting factors.

**Figure 4:**
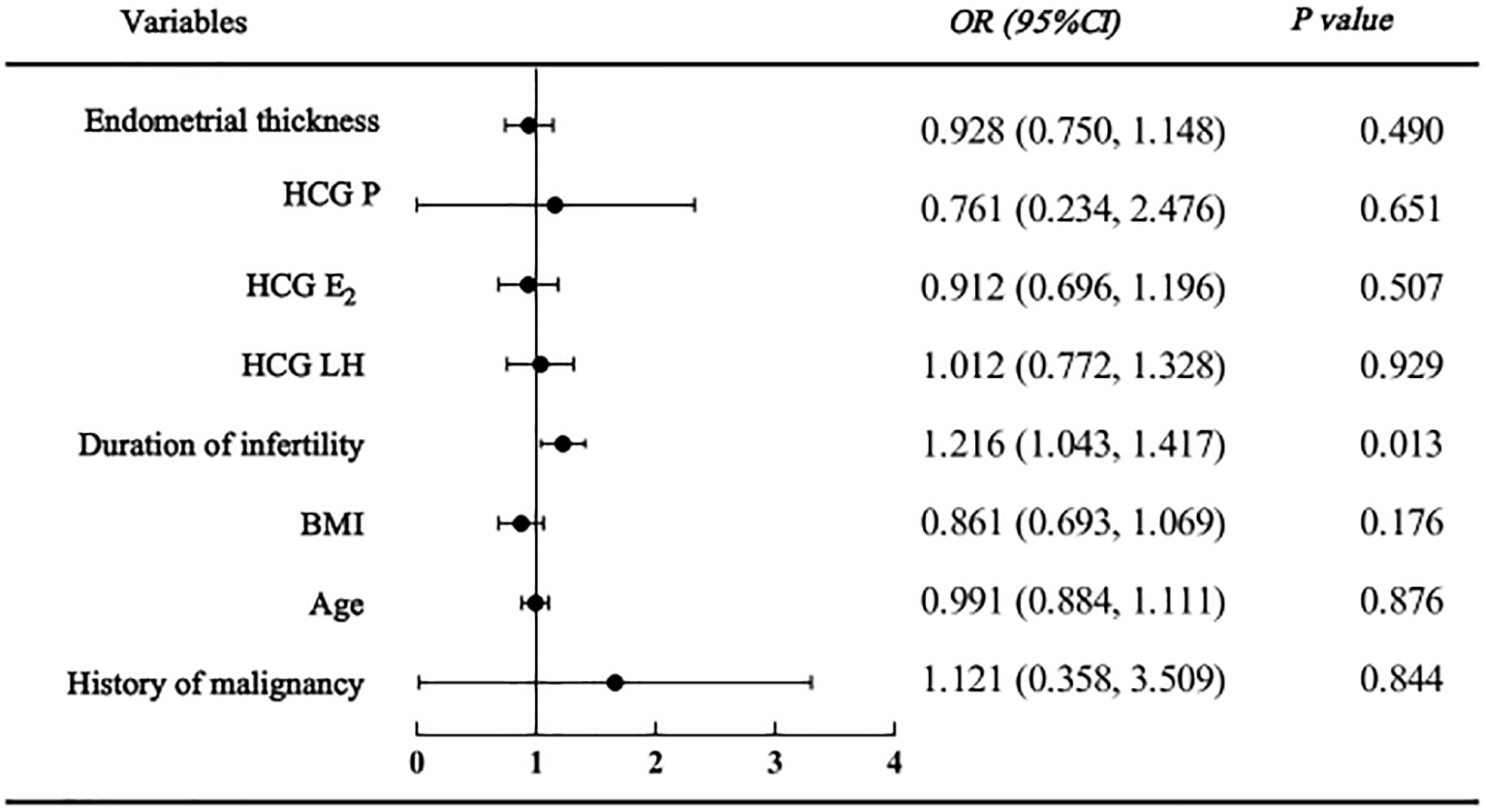
Logistic regression analysis forest plot of adverse pregnancy affecting factors.

As shown in the regression analysis, a history of malignancy was not found to be related to clinical pregnancy, live birth or adverse pregnancy outcomes. However, endometrial thickness and infertility duration might be associated with pregnancy outcomes. The clinical pregnancy model confirmed that women with a higher endometrial thickness (HR=1.134 [95% CI=1.055, 1.219], P=0.001) were more likely to become pregnant; however, those with a longer duration of infertility (HR=0.927 [95% CI=0.865, 0.994], P=0.032) were less likely to become pregnant (**Figure 2**). Similarly, in the live birth model, women with a higher endometrial thickness (HR=1.102 [95% CI 1.013, 1.018], P=0.023) were more likely to have a live birth, while those with a longer duration of infertility (HR=0.881 [95% CI=0.084, 0.965], P=0.006) were less likely to have a live birth (**Figure 3**). Furthermore, as shown by the adverse pregnancy model (**Figure 4**), infertility duration was a unique risk factor, while no statistically significant difference was observed for endometrial thickness.

#### Relationship between a history of malignancy and pregnancy outcomes

As shown in **Table 4**, a history of cancer had no effect on clinical pregnancy (HR=0.728 [95% CI 0.467, 1.135]), live birth (HR=0.824 [95% CI=0.529, 1.285]) or adverse pregnancy (OR=1.165 [95% CI=0.372, 3.649]) outcomes, and the difference was not statistically significant. However, the impact of a history of malignancy on clinical pregnancy (aHR=0.727 [95% CI=0.465, 1.136]), live birth (aHR=0.815 [95% CI=0.522, 1.274]) and adverse pregnancy (aOR=1.168 [95% CI=0.369, 3.690]) outcomes was also nonexistent when age and BMI were included in the model. Similarly, these results were confirmed when infertility duration, hormone (LH, estradiol, progesterone) levels on the hCG trigger day and endometrial thickness were included in the model.

**Table 4:** COX regression analysis results of the history of malignant tumor correlation with clinical pregnancy, live birth and adverse pregnancy.

	Clinical pregnancy*HR* (95%*CI*)	Live birth*HR* (95%*CI*)	Adverse pregnancy*OR* (95%*CI*)
History of malignancy n(%)	32 (54.24)	27 (45.76)	5 (8.47)
Non-history of malignancy n(%)	82 (69.49)	73 (61.86)	9 (7.63)
Unadjust	0.728 (0.467, 1.135)	0.824 (0.529, 1.285)	1.165 (0.372, 3.649)
Adjust^a^	0.727 (0.465, 1.136)	0.815 (0.522, 1.274)	1.168 (0.369, 3.690)
Adjust^b^	0.908 (0.562, 1.446)	0.950 (0.589, 1.534)	1.067 (0.298, 3.823)

a , adjust for age, BMI.

b , further adjust for years of infertility, HCG LH, HCG estradiol , HCG P and endometrial thickness.

BMI, body mass inde; HCG LH, Luteinizing hormone on the human chorionic gonadotropin trigger day; HCG estradiol, estradiol on the human chorionic gonadotropin trigger day; HCG P ,progesterone on the human chorionic gonadotropin trigger day.

#### Relationship between history of malignancy and pregnancy complications

In addition, we used logistic regression analysis to further explore the relationship between a the history of malignancy and pregnancy complications, suggesting that there was also no statistically significant effect of a malignancy history on pregnancy complications, including PROM, GDM, thyroid disorders in pregnancy, premature birth, and fetal macrosomia (**Table 5**). After adjustments were made for age and BMI, no significant difference remained (**Table 5**). Similarly, the impact of a history of malignancy on pregnancy complications was also nonexistent when the duration of infertility, hormone (LH, estradiol, progesterone) levels on the hCG trigger day and endometrial thickness were included in the model (**Table 5**).

**Table 5:** Logistic regression analysis results of the history of malignant tumor correlation with pregnancy complications [*OR* (95%*CI*)].

	Unadjust	Adjust^a^	Adjust^b^
Premature rupture of fetal membranes (PROM)	0.429 (0.049, 3.742)	0.443 (0.051, 3.882)	0.404 (0.034, 4.842)
Gestational diabetes mellitus(GDM)	2.365 (0.585, 9.565)	2.344 (0.573, 9.592)	3.322 (0.669, 16.497)
Thyroid disorders in Pregnancy	2.365 (0.585, 9.565)	2.527 (0.611, 10.454)	3.102 (0.706, 13.633)
Premature birth	0.577 (0.151, 2.207)	0.584 (0.152, 2.249)	0.683 (0.169, 2.765)
Fetal macrosomia	1.365 (0.119, 15.699)	1.572 (0.129, 19.104)	1.346 (0.073, 24.754)

a adjust for age, BMI.

b further adjust for years of infertility, HCG LH, HCG estradiol , HCG P and endometrial thickness.

BMI, body mass inde; HCG LH, Luteinizing hormone on the human chorionic gonadotropin trigger day; HCG estradiol, estradiol on the human chorionic gonadotropin trigger day; HCG P ,progesterone on the human chorionic gonadotropin trigger day.

### Childbirth outcome

The childbirth outcomes in the two groups were similar. There was no significant difference in the delivery method, sex of the newborns, gestational age at birth or the number of fetuses between the two groups (P>0.05) (**Table 6**). Approximately 3/4 of the patients in the cancer group or control group chose cesarean section for delivery. The mean gestational age in the cancer group was higher than that in the control group (37.98 ± 1.44 vs. 37.65 ± 2.53, P=0.528), and the mean weight (2.87 ± 0.60 vs. 2.94 ± 0.63, P=0.61) and birth height (48.12 ± 2.23 vs. 48.42 ± 2.79, P=0.579) were lower than those in the control group, but the differences were not statistically significant (**Table 6**). The probability of LGA in the cancer group was approximately 1/5 of that in the control group.

**Table 6:** Childbirth outcomes.

	Cancer group	Control group	*P-value*
**Delivery way**			
vaginal delivery	7 (25.93)	17 (23.29)	0.784
cesarean section	20 (74.07)	56 (76.71)	
**Gestational age at birth**			
Mean gestational age(SD)	37.98± 1.44	37.65 ± 2.53	0.528
premature birth	3 (11.11)	13 (17.81)	0.911
Partus maturus	24 (88.89)	60 (82.19)	
partus serotinus	-	-	
**No. fetuses**			
singleton	21 (63.64)	56 (62.22)	0.886
multiple birth	12 (36.36)	34 (37.78)	
**Sex**			
male	18 (54.55)	44 (48.89)	0.578
female	15 (45.45)	46 (51.11)	
**Birth weight**			
Mean ***weight*** (kg) (SD)	2.87±0.60	2.94±0.63	0.61
LBW	7 (21.21)	19 (21.11)	0.565
NBW	24 (72.73)	69 (76.67)	
LGA	2 (6.06)	2 (2.22)	
birth height (cm) (SD)	48.12±2.23	48.42±2.79	0.579

LBW ,low birth weight; NBW, normal birth weight; LGA, large for gestational age.

## Discussion

In recent years, the incidence of cancer has risen, patients have tended to be younger, and the cure rate of cancer is rising. Young female patients may still wish to give birth after the cancer is cured. However, the gonadotoxicity inherent to most cancer treatments induces iatrogenic infertility. For young cancer patients who wish to have children, assisted reproduction may be needed. Many studies have confirmed that pregnancy and childbirth after ART are safe and do not affect the prognosis of patients with cancer diagnosed (17–19). Thus far, however, available long-term outcome data for the population of cancer patients who undergo ART are limited, making it difficult to counsel patients with regard to the overall probability of success. Here, we conducted a retrospective study to assess the long-term reproductive outcomes of cancer patients who underwent ART treatment.

Overall, pregnancies conceived by ART among women with a history of cancer were feasible and did not seem to be detrimental to the mother or child in this retrospective cohort study. Cancer survivors showed higher preterm birth rates and low-body-weight birth rates than women in the control group, but the difference was not statistically significant, which is in accordance with previously published data (22). However, inconsistent with the results of previous studies (22, 23), there was no statistically significant difference in the number of retrieved oocytes between these two groups. Additionally, we found that a history of malignancy was not an influencing factor for pregnancy outcomes but that endometrial thickness and infertility duration might be influencing factors.

The impact of cancer on the ovarian stimulation (OS) response remains controversial. A previous studies showed that the number of retrieved oocytes was lower in women with cancer (23). However, the opposite conclusion was reached in a meta-analysis in 2018 (24). Both AFC and AMH are reliable indicators for predicting OS responses (13). The lower the AFC or AMH value is, the lower the ovarian reactivity will be. We observed a significantly lower AFC in the cancer group than in the control group (12.000 ± 7.859 vs. 14.898 ± 8.709, P=0.033), which suggests that the expected ovarian response of cancer survivors is poor. The differences in AFC, but not AMH were statistically significant in our study. Since 2006, AMH has been widely used as a routinely evaluated ovarian reserve indicator in our center. However, some of the data from before 2006 were unfortunately lacking in this study, which may explain the difference between the two indicators. Interestingly, the analysis of the first-cycle IVF/ICSI outcomes showed a lower number of oocytes retrieved in the cancer group (9 ± 7 vs. 10.144 ± 6.668, P=0.291); however, these differences did not reach statistical significance, which was unexpected because the expected ovarian response was poor in the cancer group.

Patients were enrolled for more than 10 years, during which time ART and controlled ovarian stimulation (COS) were developed. We noted significant differences in the OS protocol between the two groups (P<0.001). This may explain the unanticipated ovarian response. The antagonist protocol and mild stimulus protocol were used more frequently in the cancer group than in the age-matched control group. Letrozole was often added to the mild stimulus protocol at our center. From the present data, there was a tendency for women with a history of cancer to be treated with a low-dose Gn OS protocol and achieve a lower peak estradiol level (25). In recent years, studies have confirmed the safety and efficacy of COS with gonadotropins and letrozole, particularly in women with cancer (26). Aromatase inhibition with letrozole reduces the FSH dose required for COS (27) and maintains levels of estrogen similar to those in unstimulated cycles (28), while the number of oocytes retrieved is comparable to that of standard OS protocols. Our study showed that the cancer group received a lower total dose of gonadotrophins and had a shorter length of OS than the control group, while the numbers of retrieved oocytes were comparable between the groups. The peak estradiol level (2665.438 ± 2198.25 vs. 2980.141 ± 2458.714, P=0.134) was lower in the cancer group than in the control group; however, the difference was not statistically significant. This may be due to our insufficient sample size.

Certain cancer types affect reproductive outcomes (13). A previous study demonstrated that the main difference is that the number of mature oocytes retrieved differs among was observed between different cancer types, and is lower in patients with gynecological cancer than in those with hematological or breast cancer (3) In addition, the number of mature oocytes is related to the age of cancer onset and the prognosis of the cancer. In our study, thyroid cancer was the prevalent cancer type among the included patients. Nevertheless, according to global cancer statistics (29), breast cancer is the leading cancer in women of childbearing age. The reason for this phenomenon might be that the incidence of thyroid cancer is gradually rising globally, while the 5-year relative survival rate is generally high and exceeds 99% (30). In contrast, breast cancer is the leading cause of cancer death among females, and the onset age ranges from 30 to 39 years. Most of these patients completed assisted reproductive programs. Accordingly, the number of patients with breast cancer who underwent ART intervention was expected to increase in our study. Additionally, cancer types with different COS protocols need to be further considered in future studies.

The obstetrical outcomes of cancer patients who have achieved pregnancy after assisted reproduction have received continuous attention, but the available data are limited. To date, the published literature has reported that a history of thyroid cancer does not affect pregnancy outcomes or increase the risk of adverse obstetric outcomes after IVF/ICSI (22). Similarly, a large population-based study found that with the use of donor oocytes, the live birth rates in women with prior cancer was comparable to that of women without cancer (31). Our findings support their conclusions. We demonstrate that a history of malignancy is not a risk factor for clinical pregnancy outcomes, live birth outcomes, adverse pregnancy outcomes or pregnancy-related complications. Additionally, a similar result was found in that there was no correlation between malignancy history and pregnancy outcome after adjustments for age and BMI. Furthermore, after further adjustment for infertility duration, hormone (LH, estradiol, progesterone) levels on the hCG day and endometrial thickness, the difference was still not significant for clinical pregnancy outcomes, live birth outcomes, adverse pregnancy outcomes or pregnancy-related complications (P>0.05).

Notably, we found that endometrial thickness the duration of and infertility year were associated with pregnancy outcome. A systematic review and meta-analysis found that the mean number of oocytes retrieved and the clinical pregnancy rate in women with an endometrial thickness (EMT)≤7 mm were lower than those in women with an EMT>7 mm (P=0.001) (32). This suggests that endometrial thickness is a protective factor against conception after IVF, in line with our research. In addition, preconception factors (33), such as smoking, illicit drug use, and inadequate nutrition, reduce the potential success rates of IVF/ICSI in treating infertility. However, larger studies are needed to further confirm these observations. The likelihood of a history of cancer did not affect the pregnancy outcomes, while oocyte quality, preconception and conception factors were more important factors. Hence, continuous obstetric surveillance and close follow-up are necessary for these patients.

Large cohort studies have reported higher risks of small for gestational age (SGA) with a history of subfertility/fertility treatment, preterm birth and LBW among cancer patients (20). One possible explanation is the ART procedure itself. A meta-analysis (34) incorporating a cohort of 50 study cohorts reported a significantly increased risk of preterm birth (RR 1.71 [95% CI=1.59–1.83]; P<0.00001), LBW (RR 1.61 [95% CI=1.49–1.75]; P<0.00001), SGA (RR 1.35 [95% CI=1.20–1.52]; P<0.00001), and perinatal mortality (RR 1.64 [95% CI=1.41–1.90]; P<0.00001) in women after ART compared with those who conceived naturally. Another possible explanation is a positive result due to age, which is considered to be a major factor in the prognosis of IVF and is uncontrolled in many studies. Population-based cohort studies indicated that obstetric risk, such as gestational hypertension, eclampsia and preeclampsia, placental abruption, preterm birth, dystocia, cesarean delivery, postpartum hemorrhage, fetal growth restriction, LBW, very low birth weight, and malformation, was almost always elevated with increasing maternal age, especially in women over the age of 35 years (35). The survey results revealed that cancer patients in the ART group were older at diagnosis and at conception (36). In our study, the average age of the cancer group was 35.7 years, the average age of the control group was 35.6 years, and the difference was not statistically significant. We observed that the deliveries of women with a history of cancer after undergoing ART were not significantly different with respect to the preterm birth rate, multiple birth rate, birth weight, birth height and newborn sex compared with age-matched healthy ART patients.

This study has many strengths, including its detailed information on reproductive outcomes and long follow-up time; however, we must also recognize its limitations. Similar to other studies, our study was limited by the small sample size of its patient cohort, and we were unable to consider different types of cancers. Second, in our study, we did not assess naturally pregnant women with a history of cancer as controls. Several cohort studies have observed that female cancer survivors have lower rates of pregnancy and live birth than the general population and a higher incidence of adverse pregnancy outcomes (37) (38). Finally, there was a lack of detailed cancer treatment and tumor stage information because many cancer survivors had already completed their cancer treatment by the time when they arrived at our hospital.

## Conclusions

In summary, our findings support prior work indicating that the practice of ART is safe for women with a history of cancer. We found that a history of cancer does not decrease the number of retrieved oocytes, does not increase the risk of obstetric outcomes and does not affect birth outcomes in this patient group. However, there is a lack of consensus on the effect of a history of cancer on the safety of ART. Therefore, all patients should accept detailed counseling regarding the potential risks and require close follow-up during and after ART therapy, especially regarding the safety of the mother and child. Further research, including large-sample, multicentric studies and examinations of the long-term reproductive outcomes of cancer survivors, should be conducted to resolve these important issues.

## Data availability statement

The raw data supporting the conclusions of this article will be made available by the authors, without undue reservation.

## Ethics statement

The studies involving human participants were reviewed and approved by the Ethics Committee of Fujian Maternity and Child Health Hospital, College of Clinical Medicine for Obstetrics & Gynecology and Pediatrics, Fujian Medical University. Written informed consent for participation was not required for this study in accordance with the national legislation and the institutional requirements.

## Author contributions

YS, YL,XC and PS contributed to the conception and design of the study. HW, XY and ZK organized the database. QW performed the statistical analysis. YL and XC wrote the first draft of the manuscript. BD, BZ and AY wrote sections of the manuscript. All authors contributed to manuscript revision and read and approved the submitted version.

## Funding

This work was supported by the National Natural Science Foundation of China (Grant no. 82170908), the Natural Science Foundation of Fujian Province(Grant no.2019J01513) and the Natural Science Foundation of China (Grant no. 82003496).

## Acknowledgments

We would like to convey sincere sincerely gratitude to our colleagues at the Reproductive Medicine Center, Fujian Maternity and Child Health Hospital, College of Clinical Medicine for Obstetrics & Gynecology and Pediatrics, Fujian Medical University.

## Conflict of interest

The authors declare that the research was conducted in the absence of any commercial or financial relationships that could be construed as a potential conflict of interest.

## Publisher’s note

All claims expressed in this article are solely those of the authors and do not necessarily represent those of their affiliated organizations, or those of the publisher, the editors and the reviewers. Any product that may be evaluated in this article, or claim that may be made by its manufacturer, is not guaranteed or endorsed by the publisher.

## Abbreviations

ART, assisted reproductive technology; GnRH-a, gonadotropin-releasing hormone agonists; BMI, body mass index; FSH, follicle-stimulating hormone; LH, luteinizing hormone; Gn gonadotropin; HCG human chorionic gonadotropin; HMG human menopausal gonadotropin; AFC, antral follicle count in both ovaries; AMH, anti-Müllerian hormone; IVF, *in vitro* fertilization; ICSI intracytoplasmic sperm injection; ET, embryo transfer; OS, ovarian stimulation; COS controlled ovarian stimulation; EMT, endometrial thickness.
